# A Structure-Aware Deep Learning Framework for Automated Bridge Inspection Integrating SegFormer-Based Structural Member Segmentation and YOLOv8 Damage Detection

**DOI:** 10.3390/s26134255

**Published:** 2026-07-04

**Authors:** Sushama De Silva, Pang-jo Chun

**Affiliations:** Institute of Engineering Innovation, School of Engineering, The University of Tokyo, Tokyo 113-8656, Japan; chun@g.ecc.u-tokyo.ac.jp

**Keywords:** bridge inspection, deep learning, structural member segmentation, damage detection, SegFormer, YOLOv8, Segment Anything Model, structure-aware analysis, semantic segmentation, spatial damage mapping, damage-to-member association

## Abstract

**Highlights:**

This study presents a structure-aware deep learning framework that integrates transformer-based segmentation and object detection to automatically associate bridge damage with specific structural members, producing outputs such as “crack on main girder” providing structure-aware inspection outputs as a first layer for AI-assisted bridge inspection and subsequent maintenance assessment.

**What are the main findings?**

- The Phase 2 SegFormer model achieved a test mIoU of 0.851 using SAM mask-prompt refined annotations. Compared with the Phase 1 configuration (mIoU = 0.550), the observed improvement reflects the combined effects of SAM mask-prompt refinement, bearing-class removal, and dataset refinement rather than any single modification.
- The integrated pipeline achieved 70.0% fully correct damage detection and 62.0% fully correct member assignment on 100 previously unseen bridge inspection images, with the main girder class achieving the highest accuracy (90.9% and 93.9%, respectively).

**What are the implications of the main findings?**

- Structure-aware inspection outputs that explicitly link damage type to structural location provide more actionable information for maintenance decision-making than conventional damage-only detection approaches.
- The framework demonstrates that combining foundation model refinement (SAM) with supervised segmentation and one-stage detection is a viable and practical direction for automated infrastructure monitoring.

**Abstract:**

As a pilot-scale feasibility study, aging bridge infrastructure and limited inspection resources have created an urgent need for automated and reliable bridge condition assessment systems. Most existing deep learning-based inspection approaches detect damage types from images without considering the structural member on which the damage occurs, limiting their practical utility for maintenance decision-making. This study proposes a structure-aware deep learning framework for automated bridge inspection that integrates structural member segmentation, two-class damage detection, and spatial damage-to-member association within a unified pipeline. A SegFormer-based semantic segmentation model was trained on a custom bridge inspection dataset comprising 1339 images to identify three primary structural member classes—main girder, deck slab, and abutment—achieving a test mean Intersection over Union (mIoU) of 0.851. Boundary refinement using the Segment Anything Model (SAM) in mask-prompt mode was applied to improve mask precision during training data preparation. A YOLOv8s object detection model was trained on a custom bridge damage dataset of 9142 annotated images (6531 training, 1740 validation, and 871 test images) to detect two damage classes—crack and corrosion—achieving a mean Average Precision (mAP50) of 0.445 at a confidence threshold of 0.30. The framework associates detected damage with segmented structural members using a region-based spatial assignment strategy, enabling structure-aware outputs such as “crack on main girder” and “corrosion on deck slab.” Manual evaluation on 100 bridge inspection images demonstrated a fully correct damage detection accuracy of 70.0% and a fully correct member assignment accuracy of 62.0%. When partially correct predictions were additionally considered for qualitative analysis, the corresponding accuracies increased to 84.0% and 87.0%, respectively. The main girder class achieved the highest combined accuracy for both damage detection (90.9%) and member assignment (93.9%). These results demonstrate the potential of the proposed framework as a first layer for AI-assisted bridge inspection by associating detected damage with structural members, providing structured inspection information to support subsequent maintenance assessment and infrastructure monitoring.

## 1. Introduction

Bridges are critical components of transportation infrastructure and play a fundamental role in supporting economic activity and social connectivity. However, many bridge systems worldwide are aging and increasingly require inspection, maintenance, and rehabilitation to ensure their structural safety and serviceability. In the United States, recent infrastructure assessments report that there are more than 623,000 bridges, with approximately 45% already exceeding their original 50-year design life, while more than 221,000 bridges require repair or replacement and over 63,000 are subject to load restrictions [1]. Similar concerns exist in Japan, where bridge deterioration has become a major challenge for infrastructure management. According to the Ministry of Land, Infrastructure, Transport and Tourism (MLIT), Japan has approximately 700,000 road bridges, of which about 70–75% are managed by municipalities, and the proportion of bridges older than 50 years increased from 18% in 2013 to 43% in 2023 [2]. In addition, the number of municipal bridges with traffic restrictions has more than doubled in recent years, indicating that deterioration is already affecting serviceability and safety [2].

The challenge of aging bridges is further compounded by limitations in maintenance capacity and inspection resources. In Japan, many municipalities are responsible for a large number of bridges but often lack sufficient engineering personnel for bridge maintenance. MLIT data indicate that 50% of towns and 70% of villages do not have civil engineering technicians dedicated to bridge maintenance, while some municipal inspection practices still rely on distant visual inspection, which may lead to unreliable condition assessment [2]. Similar infrastructure management challenges are also observed in developing countries. In Sri Lanka, for example, more than 4200 bridges are managed under the national road authority, and many older bridges have been identified as requiring rehabilitation, reconstruction, or load capacity enhancement [3]. These trends demonstrate that aging bridge infrastructure is not only a national issue but also a global engineering problem.

Traditionally, bridge condition assessment has relied primarily on manual visual inspection. Although visual inspection remains the most common method because of its simplicity and practical applicability, it is labor-intensive, costly, time-consuming, and strongly dependent on the experience and judgment of inspectors [4,5]. In some cases, bridge inspectors must access high or difficult-to-reach locations using rope access systems or high-elevation work platforms, which increases both cost and safety risk [5]. Moreover, inadequate inspection and condition assessment may lead to serious failures. For example, the collapse of the I-35W Highway Bridge in Minneapolis in 2007 resulted in 13 fatalities and 145 injuries, and investigation reports highlighted shortcomings in inspection guidance and condition assessment practices [4]. Such cases emphasize the importance of more reliable and efficient inspection technologies.

To address these limitations, computer vision, machine learning, and deep learning techniques have been increasingly investigated for bridge inspection and structural health monitoring. Early studies demonstrated that computer vision-based methods could support automated defect detection and condition assessment of civil infrastructure [4]. More recent advances in deep learning, especially convolutional neural networks (CNNs), have significantly improved automated crack detection and damage recognition performance [6,7]. In addition, the rapid development of unmanned aerial vehicles (UAVs) has enabled inspectors to collect high-resolution visual data from difficult-to-access bridge components, supporting safer and more efficient non-contact inspection workflows [5,8]. Recent research has further explored hybrid frameworks combining UAV imagery and deep learning models to improve structural health monitoring under real-world field conditions [8]. More recently, transformer-based segmentation architectures such as SegFormer [9] and foundation models such as the Segment Anything Model (SAM) [10] have opened new opportunities for precise structural component delineation and boundary refinement in complex visual scenes.

Despite these advancements, most existing studies still focus primarily on detecting damage types from images without sufficiently considering the structural member on which the damage occurs. In practical bridge maintenance, engineers must interpret not only the type of damage, but also its structural location, since the same damage type may have different implications depending on whether it appears on a main girder, deck slab, abutment, or other bridge component [11]. Some recent studies have begun to address bridge components and damage jointly. For example, multilevel structural component detection and segmentation methods have been proposed for computer vision-based bridge inspection [12], and bridge damaged-object detection frameworks using bridge member models have also been reported [13]. In addition, recent multiclass bridge surface damage detection methods have demonstrated promising results for identifying and segmenting various damage categories across bridge components [11]. However, the integration of structural member segmentation, multiclass damage detection, and spatial damage-to-member association within a unified framework for bridge inspection remains limited. The present study addresses automated visual inspection—the use of image-based deep learning to detect and localize surface damage from bridge inspection photographs—rather than structural health monitoring in the strict sensor-based sense, which typically involves temporal physical measurements such as vibration, strain, or acoustic data.

Motivated by this gap, this study proposes a structure-aware deep learning framework for automated bridge inspection that integrates SegFormer-based structural member segmentation, YOLOv8-based multiclass damage detection, and a spatial damage mapping strategy. In the proposed framework, structural members are first identified using a transformer-based segmentation model refined with SAM-assisted mask preparation. Damage types are then detected from bridge inspection images using an efficient one-stage detection model, and the detected damage regions are spatially associated with the corresponding structural members. By combining these stages, the framework produces structure-aware outputs that provide more informative and actionable results for practical bridge condition assessment and maintenance decision-making.

The main contributions of this study are summarized as follows:

- Development of a structure-aware bridge inspection framework that integrates transformer-based structural member segmentation with one-stage damage detection and region-based spatial assignment, producing labeled outputs such as “crack on main girder” and “corrosion on deck slab” that provide structural context for maintenance decision-making as part of a broader inspection workflow—a capability not demonstrated by existing damage-only or segmentation-only approaches.
- Application of SAM mask-prompt refinement for ground-truth annotation improvement in structural member segmentation, contributing to a Phase 2 test mIoU of 0.851. The observed improvement over the Phase 1 configuration (mIoU = 0.550) reflects the combined effects of annotation refinement, bearing-class removal, and dataset refinement rather than any single methodological change.
- Implementation of a spatial damage mapping strategy that associates detected damage with specific structural members, enabling structure-aware inspection outputs.
- Systematic manual evaluation of the integrated pipeline on 100 previously unseen bridge inspection images, demonstrating fully correct damage detection and member assignment accuracies of 70.0% and 62.0%, respectively, with per-member-class performance analysis.

## 2. Literature Review

### 2.1. Bridge Inspection and Infrastructure Deterioration

Bridges are essential components of transportation infrastructure and play a vital role in maintaining connectivity within transportation networks. However, many bridges worldwide are approaching or exceeding their design service life, which increases the risk of structural deterioration and potential safety issues. Structural deterioration may occur due to environmental exposure, repeated loading, corrosion, and natural hazards, making regular inspection and maintenance essential for ensuring structural reliability and public safety [1,2,3].

Traditionally, bridge inspection relies primarily on manual visual inspection performed by trained engineers. Although visual inspection is widely used due to its practicality, it is often labor-intensive, time-consuming, and highly dependent on the experience and judgment of inspectors. In many cases, inspectors must access hazardous locations such as high bridge decks or underside components using specialized equipment, which increases inspection cost and risk [4,5].

To address these limitations, researchers have explored advanced inspection technologies such as unmanned aerial vehicles (UAVs), remote sensing systems, and automated monitoring techniques. UAV-based bridge inspection systems enable efficient image acquisition from difficult-to-access locations and significantly improve inspection safety and efficiency. The integration of UAV platforms with artificial intelligence and remote sensing technologies has therefore been increasingly investigated for infrastructure inspection applications [5,8].

### 2.2. Deep Learning for Damage Detection

Recent advances in deep learning have significantly improved automated damage detection in civil infrastructure systems. Convolutional neural networks (CNNs) have demonstrated strong capability in extracting complex image features and identifying structural defects such as cracks, corrosion, and surface deterioration in bridge components [6,14,15].

Earlier studies on crack detection relied mainly on traditional image processing techniques such as edge detection, thresholding, and morphological filtering. However, these methods often struggled to achieve reliable performance under varying lighting conditions, surface textures, and environmental noise [16].

With the development of deep learning methods, CNN-based models have shown improved performance in detecting cracks and other structural defects from image data. For example, hierarchical convolutional networks have been proposed to learn multi-scale crack features and accurately detect crack patterns in structural images [6]. Similarly, deep learning frameworks have been developed to detect multiple types of structural damage in infrastructure inspection images using advanced convolutional architectures [15,17]. More recently, YOLOv8-based approaches have further advanced automated bridge damage detection through enhanced feature extraction and optimized detection architectures, demonstrating superior performance in bridge defect detection [18,19,20]. These advances have also enabled multiclass bridge surface damage detection, demonstrating the ability to identify and localize multiple damage categories simultaneously across bridge components [11].

Despite these advances, many existing deep learning-based approaches focus mainly on identifying damage types within images without explicitly considering the structural component where the damage occurs. In practical bridge inspection, engineers must interpret both the type of damage and its structural location in order to determine appropriate maintenance strategies.

### 2.3. Structural Member Segmentation in Infrastructure Inspection

In addition to damage detection, identifying structural components within bridge images is important for accurate structural assessment. Bridge structures consist of multiple structural members such as girders, decks, piers, bearings, and bracing systems, and each component plays a different role in structural performance and maintenance planning.

Recent developments in computer vision have enabled automated structural component recognition using deep learning-based segmentation techniques. These approaches allow image analysis systems to identify and classify structural members within complex bridge scenes, enabling more detailed infrastructure inspection and analysis [12]. Furthermore, deep learning-based frameworks that combine structural member recognition with damage detection have been proposed to improve the practical applicability of automated inspection systems [13].

More recently, transformer-based architectures have demonstrated superior performance in semantic segmentation tasks compared to conventional CNN-based methods. SegFormer, proposed by Xie et al., combines a hierarchical transformer encoder with a lightweight multilayer perceptron decoder, enabling efficient multi-scale feature extraction without positional encoding, which allows the model to generalize across varying image resolutions [9]. These advances have motivated the application of transformer-based segmentation models to infrastructure inspection tasks, where accurate delineation of structural components from diverse viewpoints and imaging conditions is required.

In parallel, foundation models for image segmentation have emerged as powerful tools for boundary refinement and general-purpose segmentation. The Segment Anything Model (SAM), introduced by Kirillov et al., demonstrated strong zero-shot segmentation capability across diverse image domains through a promptable architecture that accepts point, box, and mask inputs [10]. Recent studies have explored the application of SAM in specialized inspection and remote sensing contexts, leveraging its boundary precision to refine coarse segmentation outputs. However, the integration of SAM with supervised structural segmentation models for bridge member delineation remains underexplored. In particular, no prior study has systematically evaluated different SAM prompting strategies—specifically bounding-box versus mask-prompt modes—in the context of structural member segmentation for bridge inspection, nor has the comparative effect of these prompting strategies on downstream damage-to-member association accuracy been investigated. Recent transformer-based bridge inspection studies [21,22] demonstrate the potential of these architectures but do not investigate different SAM prompting strategies for structural member segmentation or downstream damage-to-member association.

### 2.4. Research Gap

Although significant progress has been made in automated bridge inspection using deep learning techniques, several limitations remain in existing research. Most current studies focus primarily on detecting damage types such as cracks or corrosion using image-based deep learning models. However, these approaches generally analyze damage independently of the structural component where it occurs.

In practical bridge inspection, engineers must evaluate both the type of damage and the structural member affected by the damage, as different structural components require different inspection criteria and maintenance strategies. For example, damage occurring on a primary load-carrying member may have different structural implications compared to similar damage on a secondary component.

Furthermore, existing segmentation approaches for bridge structural members often produce coarse pixel-level boundaries, particularly in regions with shadow, occlusion, or low texture contrast. Traditional semantic segmentation models trained on limited inspection datasets may struggle to precisely delineate structural member boundaries under such conditions. This limitation can reduce the reliability of downstream damage-to-member association, as imprecise masks may lead to incorrect structural assignments. A boundary refinement mechanism capable of improving mask precision without requiring additional manual annotation is therefore needed to support accurate structure-aware inspection.

Therefore, there is a clear need for an integrated framework that combines structural member segmentation, two-class damage detection, and spatial damage-to-member association within a unified deep learning pipeline. Such a framework would allow automated inspection systems to produce structure-aware outputs—such as “crack on main girder” or “corrosion on deck slab”—that directly support maintenance assessment based on both damage type and structural location. Bridge structural health monitoring studies have also demonstrated the importance of member-level assessment for damage identification and condition evaluation [23,24,25,26]. The present study addresses this gap by proposing an integrated pipeline that combines transformer-based structural member segmentation, foundation-model-assisted mask refinement, and one-stage damage detection with spatial member assignment, and by providing a per-member-class quantitative evaluation of structure-aware outputs on real bridge inspection imagery.

## 3. Methodology

Automated bridge inspection requires accurate identification of structural components and reliable detection of structural damage within complex inspection images. To address this challenge, this study proposes a deep learning-based framework that integrates structural member segmentation, damage detection, and structure-aware damage analysis within a unified inspection pipeline.

**Figure 1:**
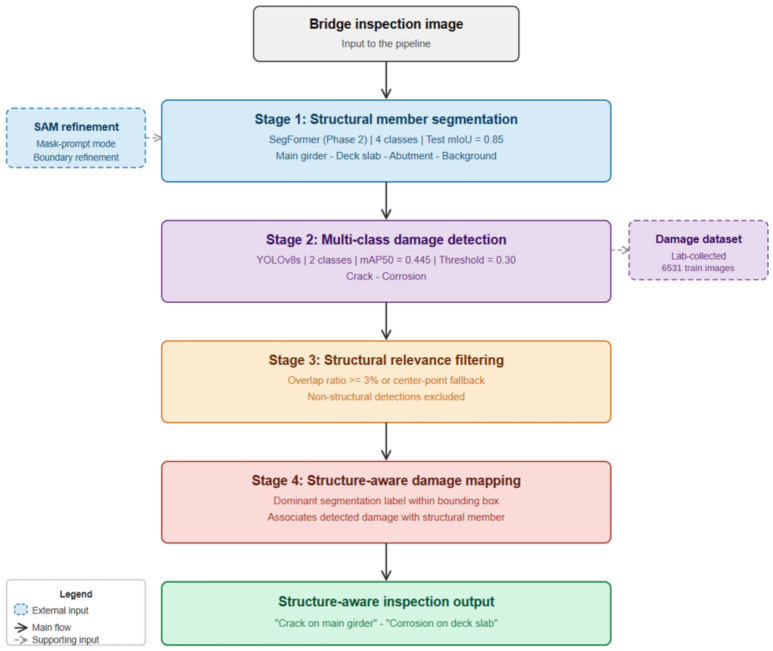
Overall workflow of the proposed bridge inspection framework integrating structural member segmentation, damage detection, and structure-aware damage mapping.

The overall workflow of the proposed framework is illustrated in Figure 1. The framework processes bridge inspection images through a sequence of stages designed to extract structural information and associate detected damage with specific bridge components.

First, bridge inspection images are processed to identify the structural members present in the scene. A semantic segmentation model based on the SegFormer architecture is used to segment major bridge components such as main girders, deck slabs, and abutments. This stage provides the structural context of the bridge and enables the system to understand the spatial relationships between different structural elements.

Next, a damage detection model is applied to identify different types of structural damage within the inspection images. The detection stage locates potential damage regions, such as cracks and corrosion, using a deep learning-based object detection approach.

To ensure structural relevance, the detected damage regions are evaluated with respect to the segmented structural members. Detections that do not correspond to the target structural components are excluded from further analysis.

Finally, the valid damage detections are associated with the segmented bridge structural members. This structure-aware damage mapping step allows the framework to determine which specific bridge component contains the detected damage, enabling more informative and practical inspection outputs for infrastructure monitoring. Based on this workflow, the proposed methodology is organized into four main stages:

1. Structural Member Segmentation (SegFormer): Extraction of bridge structural components from inspection images using a transformer-based semantic segmentation model. Major structural members, including main girders, deck slabs, and abutments, are identified to provide structural context.
2. Multi-Class Damage Detection: Detection of different types of structural damage present in bridge inspection images using a deep learning-based object detection model. Damage types considered include cracks and corrosion.
3. Structural Relevance Filtering: Filtering of detected damage regions based on their spatial correspondence with segmented structural members. Detections associated with non-structural elements are excluded to ensure structure-focused analysis.
4. Structure-Aware Damage Mapping: Association of validated damage detections with specific bridge structural members to support structure-based inspection and assessment.

The detailed methodology for each stage of the proposed framework is described in the following sections.

### 3.1. Structural Member Segmentation Using SegFormer

The first stage of the proposed framework focuses on identifying bridge structural members from inspection images using a semantic segmentation approach. Accurate segmentation of structural components is essential for providing structural context in subsequent damage detection and analysis stages.

In this study, a semantic segmentation model based on the SegFormer architecture [9] was employed to extract major bridge structural components from inspection images. SegFormer is a transformer-based segmentation model that combines hierarchical transformer encoders with a lightweight multilayer perceptron decoder, enabling efficient extraction of both local and global contextual features in complex visual scenes.

#### 3.1.1. Bridge Inspection Image Dataset

The bridge inspection images used in this study were obtained from a custom dataset collected during routine bridge inspection activities. These images represent real inspection environments and include structural components such as main girders, deck slabs, abutments, and other bridge elements. Images were captured using standard inspection cameras, and the dataset reflects the visual diversity typically encountered in field inspection conditions. The final Phase 2 dataset contains 1092 images, split into 945 training, 74 validation, and 73 test images. This 1092-image dataset represents the final Phase 2 training subset obtained after quality control and class harmonization of the fully integrated segmentation dataset described in Section 3.1.3, which contains 1339 images. The final 1092-image Phase 2 dataset was derived from the 1339-image integrated segmentation dataset after quality control and class harmonization. The excluded images did not satisfy the final Phase 2 class harmonization criteria.

To ensure high-quality annotations for segmentation tasks, images were carefully selected based on several criteria. Images containing people, ultraviolet inspection markings, or excessive darkness were excluded to avoid interference with the annotation process. Images with clear visibility of structural members were prioritized in order to facilitate accurate pixel-level labeling. Using this selection process, a total of 804 bridge inspection images were prepared for annotation.

#### 3.1.2. Pixel-Level Annotation and SAM-Assisted Boundary Refinement

To generate ground-truth segmentation masks, the selected bridge inspection images were manually annotated using the Computer Vision Annotation Tool (CVAT, version 2.69.0). CVAT is an open-source annotation platform widely used for preparing datasets for computer vision and deep learning applications. The tool provides polygon-based annotation capabilities that allow precise delineation of object boundaries within images.

During the annotation process, each image was labeled with polygon masks corresponding to bridge structural components and surrounding contextual elements. Among the 804 images prepared for annotation, 743 images contained identifiable structural components and were successfully annotated, while 61 images were excluded due to insufficient structural information. The annotation process produced a total of 2036 segmentation masks across multiple structural and non-structural classes. Structural classes included STR_main_girder, STR_deck_slab, STR_abutment, and STR_cross_beam. In addition, contextual elements such as guard rails, drainage pipes, and parapets were annotated as non-structural classes.

To further improve mask boundary quality, the Segment Anything Model (SAM) was applied as a post-processing refinement step on the initial segmentation masks. SAM was used in mask-prompt mode, where the coarse segmentation predictions served as spatial priors to guide SAM in generating refined pixel-level boundaries. This hybrid approach enabled more precise delineation of structural member boundaries, particularly for large continuous members such as main girders and deck slabs, without requiring additional manual re-annotation. The refined masks were used as the final ground-truth annotations for model training.

#### 3.1.3. Dataset Integration and Preparation

To improve training robustness and increase the diversity of structural samples, the annotated dataset was integrated with an additional bridge member mask dataset containing previously generated segmentation masks. Before integration, class labels of the two datasets were harmonized to ensure consistency with the structural annotation scheme adopted in this study. Only classes relevant to bridge structural components were retained for segmentation training. The integrated dataset contains segmentation masks for three primary bridge structural members: STR_main_girder, STR_deck_slab, and STR_abutment.

The combined dataset was divided into training, validation, and testing subsets to enable reliable model training and evaluation. The primary dataset was split using a 70–15–15% ratio, resulting in 520 training images, 111 validation images, and 112 test images. The supplementary dataset followed an 80–10–10% split, producing 476 training images, 59 validation images, and 61 test images. After integration, the combined dataset used for segmentation preparation consisted of 1339 bridge inspection images, comprising 996 training, 170 validation, and 173 test images. This integrated dataset was created after dataset integration and class harmonization and served as the basis for subsequent Phase 2 quality control. The final 1092-image Phase 2 training dataset used for SegFormer training was derived from this integrated dataset, as described in Section 3.1.1. Training, validation, and test datasets were mutually exclusive with no overlapping images. Furthermore, the 100 bridge inspection images used for manual evaluation were selected exclusively from the held-out test data and were not included in the training or validation sets of either the SegFormer segmentation model or the YOLOv8 damage detection model.

#### 3.1.4. SegFormer Model Configuration and Training

The SegFormer model training was conducted in two phases. In Phase 1, five classes were used, including background, STR_main_girder, STR_deck_slab, STR_abutment, and STR_bearing. However, the bearing class consistently achieved zero IoU throughout training due to its small spatial extent and limited representation in the dataset. Based on this observation, the bearing class was excluded in Phase 2 to reduce class imbalance and improve overall training stability. Both phases used the SegFormer-B2 backbone (pretrained on ImageNet-1K), AdamW optimizer with learning rate 6 × 10^−5^, weight decay 0.01 (Phase 1)/1 × 10^−4^ (Phase 2), batch size 4, image resolution 512 × 512 pixels, and 20 training epochs per phase. Best model checkpoint: epoch 10 in Phase 1 (val mIoU = 0.597) and epoch 16 in Phase 2 (val mIoU = 0.822). Training was performed using Google Colaboratory (Google LLC, Mountain View, CA, USA) on an NVIDIA Tesla T4 GPU (NVIDIA Corporation, Santa Clara, CA, USA) with a random seed of 42 (Phase 1). The software environment comprised Python 3.12.13, PyTorch 2.11.0 (CUDA 12.8), Torchvision 0.26.0, Transformers 5.12.1, NumPy 2.0.2, and OpenCV 4.13.0.

In Phase 2, the model was trained on four classes: background, STR_main_girder, STR_deck_slab, and STR_abutment. This configuration enables the model to focus on the primary load-bearing structural components most relevant to bridge inspection and structural condition assessment. By leveraging the transformer-based architecture of SegFormer, the model effectively captures spatial relationships between structural members and surrounding regions in complex bridge inspection scenes.

The final Phase 2 model achieved a test mean Intersection over Union (mIoU) of 0.85. Per-class IoU values on the test set were 0.92 for background, 0.84 for STR_main_girder, 0.80 for STR_deck_slab, and 0.84 for STR_abutment. Table 1 summarizes the segmentation performance across the two training phases and improvement configurations evaluated during model development.

A stability check evaluating the model across training, validation, and test splits confirmed consistent generalization behavior, with training mIoU of 0.93 and validation and test mIoU values of 0.78 and 0.85 respectively. The close agreement between validation and test performance indicates no evidence of data leakage or overfitting. The segmentation outputs generated by this stage provide the structural context required for the subsequent damage detection and structure-aware damage analysis stages of the proposed framework.

### 3.2. Two-Class Bridge Damage Detection

The second stage of the proposed framework focuses on identifying structural damage within bridge inspection images using an object detection approach. Accurate detection of damage regions is essential for assessing structural condition and supporting maintenance decision-making. In this study, a deep learning-based object detection model based on the YOLOv8 architecture [18] was employed to detect and localize structural damage. YOLOv8 is a one-stage detection framework that performs object localization and classification simultaneously, enabling efficient and real-time performance. Its capability to process entire images in a single forward pass makes it well suited for large-scale bridge inspection applications.

#### 3.2.1. Damage Detection Dataset

The structural member segmentation and damage detection models were trained independently using separate datasets and annotation schemes. The segmentation dataset consisted of pixel-level annotations of bridge structural members (main girder, deck slab, and abutment), whereas the damage detection dataset consisted of bounding-box annotations for crack and corrosion defects. No image-level pairing between the two datasets was required during model training, as the two models were developed independently and integrated only during the final structure-aware damage mapping stage.

The damage detection model was trained using a custom bridge damage dataset collected under real inspection conditions, containing annotated images of bridge surfaces exhibiting various types of deterioration. Three model configurations were evaluated during development: a three-class baseline model detecting crack, corrosion, and leakage; a three-class model with augmented leakage training data; and a final simplified two-class model detecting crack and corrosion only.

The leakage class consistently showed the weakest detection performance across all configurations. Even after targeted data augmentation that expanded leakage training samples from 321 to approximately 1000 images, the leakage mAP50 remained low at 0.145, compared to 0.313 in the baseline. This result indicates that the detection difficulty for leakage is primarily due to intrinsic visual variability—including irregular boundaries, low contrast, and high similarity to background staining—rather than insufficient training data. Based on this analysis, the leakage class was excluded from the final model configuration to improve detection reliability and training stability.

The final two-class damage dataset comprised 9142 annotated images, which were divided into 6531 training images, 1740 validation images, and 871 test images. The dataset provides diverse examples of structural damage under varying lighting conditions, viewpoints, and surface textures, enabling robust model training. Across all dataset splits, the annotations include 6818 crack instances and 11,879 corrosion instances. Unless otherwise stated, references to 6531 images in this study refer specifically to the training subset rather than the complete dataset.

#### 3.2.2. Annotation Format and Data Preparation

Damage annotations were provided in YOLO format, where each image is associated with a corresponding text file containing normalized bounding box coordinates. Each annotation specifies the damage class and the spatial location of the damage region. Prior to training, the dataset was verified to ensure consistency between images and labels. Corrupted samples and mismatched annotations were removed to improve data quality and training reliability.

#### 3.2.3. YOLOv8 Model Configuration and Training

The YOLOv8s model was selected as the base architecture for damage detection due to its balance between accuracy and computational efficiency. The model was trained using the following configuration: input image size of 640 × 640 pixels, batch size of 8, 40 training epochs, and 2 data loading workers. The model was optimized to detect crack and corrosion instances by learning both spatial and visual characteristics of damage patterns. During training, the model minimizes localization and classification errors to improve detection accuracy. Training configuration: 40 epochs, batch size 8, image resolution 640 × 640, SGD optimizer with lr_0_ = 0.01, momentum = 0.937, weight decay = 0.0005, 3 warmup epochs, NMS IoU = 0.70, seed = 0. Augmentation: mosaic (1.0), HSV jitter, horizontal flip (*p* = 0.5), random erasing (0.4). Pretrained COCO weights on NVIDIA T4 GPU via Google Colab.

#### 3.2.4. Damage Detection Performance

The final two-class YOLOv8s model achieved a Precision of 0.536, Recall of 0.427, mAP50 of 0.445, and mAP50–95 of 0.235 on the test dataset. The F1 score, computed as the harmonic mean of Precision and Recall, was 0.474 overall (crack: 0.523; corrosion: 0.387), reflecting moderate detection balance between the two classes. Class-wise mAP50 was 0.552 for crack and 0.339 for corrosion. Table 2 summarizes the comparative performance across the three model configurations evaluated during development.

The removal of the leakage class improved overall Precision and mAP50, while crack detection performance remained stable across configurations (mAP50 of 0.556 in the baseline versus 0.552 in the final model). A confidence threshold of 0.30 was selected for inference based on qualitative comparison at thresholds of 0.15, 0.30, and 0.50. A threshold of 0.15 produced excessive false positives, while 0.50 resulted in missed detections of smaller or lower-contrast damage regions. A threshold of 0.30 provided the best balance between detection sensitivity and reliability and was therefore adopted for all subsequent analysis.

#### 3.2.5. Damage Detection Output

The trained model outputs bounding boxes, class labels, and confidence scores for each detected damage instance. These outputs provide spatial localization of damage regions within bridge inspection images. Although bounding boxes represent approximate damage regions rather than precise pixel-level boundaries, they provide sufficient spatial information for subsequent structure-aware analysis. The detected damage regions are further processed in the following stages to ensure structural relevance and enable mapping to specific bridge components.

### 3.3. Structural Relevance Filtering

Note: SAM is applied exclusively during offline training data preparation and is not part of the inference pipeline; the inference pipeline consists solely of SegFormer and YOLOv8s. Since object detection models may identify damage on both structural and non-structural elements, a filtering mechanism is introduced to retain only structurally meaningful detections. In this study, the spatial relationship between detected damage regions and segmented structural members is evaluated. Damage detections that do not sufficiently correspond to the target structural components—main girders, deck slabs, and abutments—are classified as non-structural and excluded from further analysis. This filtering step improves the reliability of the proposed framework by ensuring that only damage associated with key load-bearing structural members is considered in the subsequent stage. This step is particularly important given that the YOLOv8s model may detect damage-like patterns on non-structural elements such as signage, vegetation, or equipment visible in field inspection images. The structural relevance filter acts as a gating mechanism that improves the precision of structure-aware outputs by suppressing detections that, while potentially valid as damage instances, are not associated with the primary load-bearing members under inspection.

### 3.4. Structure-Aware Damage Analysis

The final stage of the proposed framework integrates the outputs from structural member segmentation and damage detection to perform structure-aware damage analysis. This step enables the association of detected damage with specific bridge structural components, thereby improving the interpretability and practical relevance of the inspection results.

For each detected damage instance, represented by a bounding box obtained from the YOLOv8 model (Section 3.2), its spatial relationship with the segmented structural member masks produced by the SegFormer model (Section 3.1) is evaluated using a two-step spatial assignment strategy.

In the first step, the distribution of segmentation labels within the damage bounding box region is analyzed. The proportion of pixels belonging to each structural class within the bounding box is computed, and if the dominant structural class occupies at least 3% of the bounding box area, that class is assigned as the corresponding structural member for the detected damage. The 3% overlap threshold was selected empirically during pipeline development after evaluating more conservative thresholds of 15% and 8%. Higher thresholds frequently failed to assign small or elongated damage regions to their corresponding structural members, whereas the 3% threshold provided a better balance between assignment robustness and avoidance of false structural associations. This region-based approach is conceptually related to overlap-based methods such as Intersection over Union (IoU), but is specifically adapted for the integration of object detection bounding boxes with dense semantic segmentation masks.

In cases where no structural class meets the minimum overlap threshold, a center-point fallback strategy is applied. The structural label at the geometric center of the damage bounding box is retrieved from the segmentation mask and used for member assignment. This fallback improves robustness in cases where the damage region spans multiple structural and non-structural areas, or where the segmented member occupies only a small portion of the bounding box.

Detections for which neither the overlap criterion nor the center-point fallback yields a valid structural member assignment are classified as belonging to an unknown or non-structural region and excluded from the final inspection output.

This process enables the generation of structure-aware inspection results, such as “crack on main girder” or “corrosion on deck slab”, which provide more meaningful and actionable information compared to conventional damage detection approaches that report damage type alone without considering structural context.

For qualitative validation of the integrated framework, 100 images were manually selected from an independent bridge inspection dataset that had not been used during model development, training, validation, or testing. Image selection was performed prior to model inference based on practical inspection considerations, including image quality, lighting conditions, visibility of structural members, and the presence of the target damage classes (crack and corrosion). The finalized integrated pipeline was subsequently applied to these previously unseen images, and the predicted damage type and associated structural member were manually compared with visual inspection records. Evaluation was conducted at the image level, and images containing multiple damage instances were assessed using the complete structure-aware output generated for each image.

## 4. Results

### 4.1. Structural Member Segmentation Results

The SegFormer-based structural member segmentation model was evaluated across two training phases to identify the optimal configuration for the proposed framework. Table 1 summarizes the segmentation performance across all evaluated configurations.

In Phase 1, the model was trained on five classes including STR_bearing. The baseline SegFormer model achieved a test mIoU of 0.611. Application of SAM in bounding-box prompt mode decreased overall performance to 0.550, indicating that bounding-box prompts alone were insufficient to capture precise structural boundaries. SAM mask-prompt refinement partially recovered performance to 0.614; however, the STR_bearing class consistently achieved an IoU of 0.000 across all Phase 1 configurations due to its small spatial extent and limited representation in the training data.

Based on these observations, the bearing class was excluded in Phase 2, and the model was retrained using SAM mask-prompt refined annotations on four classes. This configuration produced substantially improved results, achieving a validation mIoU of 0.822 and a test mIoU of 0.851. Per-class test IoU values were 0.917 for background, 0.843 for STR_main_girder, 0.802 for STR_deck_slab, and 0.843 for STR_abutment. The observed improvement from Phase 1 (mIoU = 0.611) to Phase 2 (mIoU = 0.851) followed the introduction of three simultaneous methodological changes: removal of the bearing class, dataset refinement through quality control and class harmonization, and improved annotation quality using SAM mask-prompt refinement. Because these modifications were introduced concurrently, the individual contribution of each component cannot be isolated in the present study.

The comparative segmentation performance across all evaluated configurations is summarized in Table 1 (Section 3.1.4). A stability check evaluating training, validation, and test performance confirmed consistent generalization behavior, with training, validation, and test mIoU values of 0.931, 0.784, and 0.851, respectively. The close agreement between the validation and test mIoU values indicates no evidence of data leakage or overfitting, while the higher training performance is expected given the smaller held-out dataset and the greater structural and viewpoint variability present in the validation and test images.

**Figure 2:**
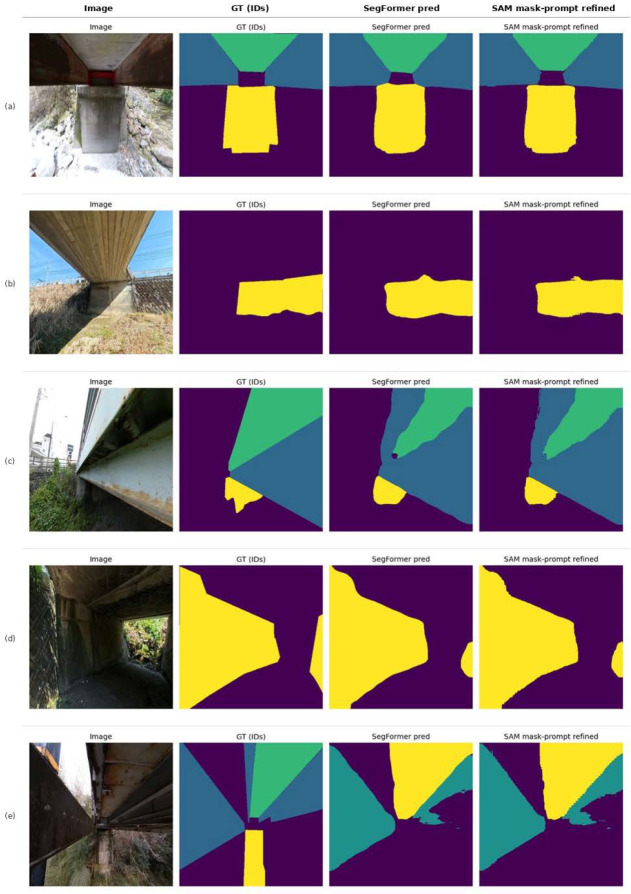
Representative segmentation results from the Phase 2 SegFormer model on test images. (**a**) Bridge scene containing the three structural member classes (main girder, deck slab, and abutment), illustrating improved boundary refinement after SAM mask-prompt refinement. (**b**) Deck slab segmentation example showing close agreement between the refined prediction and the ground truth. (**c**) Oblique bridge view demonstrating improved delineation of multiple structural members following SAM mask-prompt refinement. (**d**) Large structural component segmentation example showing accurate prediction for a dominant deck slab region. (**e**) Complex bridge scene containing multiple structural members, illustrating improved segmentation consistency after SAM mask-prompt refinement. Colors represent: purple = background, yellow = STR_main_girder, teal = STR_deck_slab, and green = STR_abutment.

Figure 2 illustrates representative qualitative results from the Phase 2 segmentation model, showing the original bridge inspection image, the ground-truth mask, the SegFormer prediction, and the SAM mask-prompt refined output for selected test samples. The model correctly delineates major structural members including main girders, deck slabs, and abutments across diverse viewpoints and imaging conditions. Minor discrepancies are primarily observed at structural boundaries and in shadowed or occluded regions, which is consistent with the quantitative results.

### 4.2. Damage Detection Results

The YOLOv8s damage detection model was evaluated across three configurations to determine the optimal class structure and training strategy. The comparative results are summarized in Table 2.

The three-class baseline model, trained to detect crack, corrosion, and leakage, achieved a Precision of 0.482, Recall of 0.423, and mAP50 of 0.409. Class-wise evaluation showed strong performance for crack detection (mAP50 = 0.556), moderate performance for corrosion (mAP50 = 0.358), and consistently weak performance for leakage (mAP50 = 0.313). To address leakage detection limitations, a targeted augmentation strategy was applied, expanding leakage training samples from 321 to approximately 1000 images through brightness adjustment, slight rotation, blur, and mild scaling. Despite this augmentation, leakage detection performance further decreased to an mAP50 of 0.145, confirming that the difficulty is attributable to the intrinsic visual complexity of leakage patterns—including irregular boundaries, diffuse staining, and high visual similarity to background surfaces—rather than insufficient training data.

**Figure 3:**
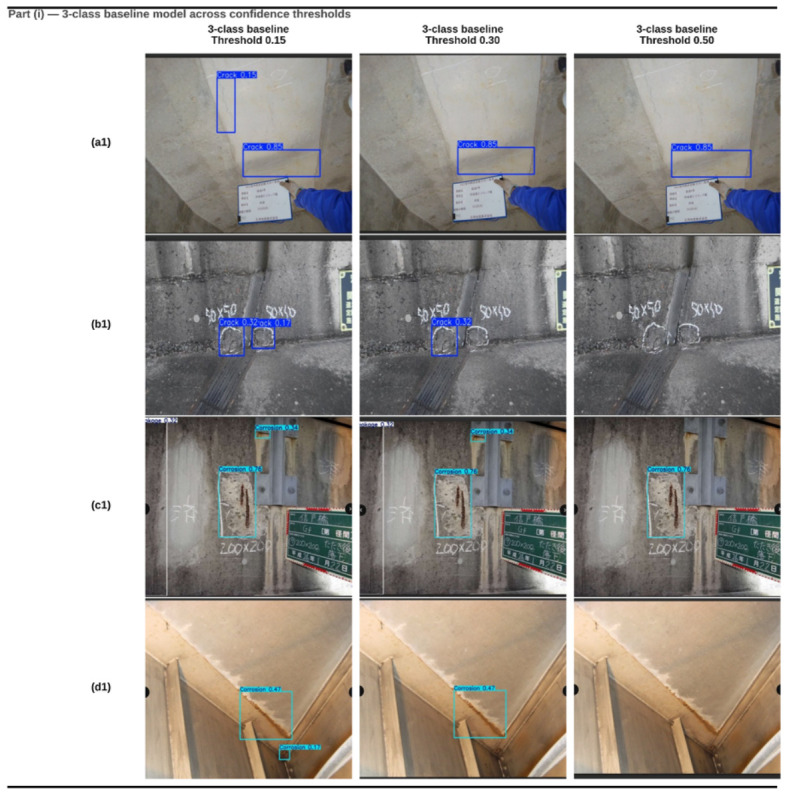

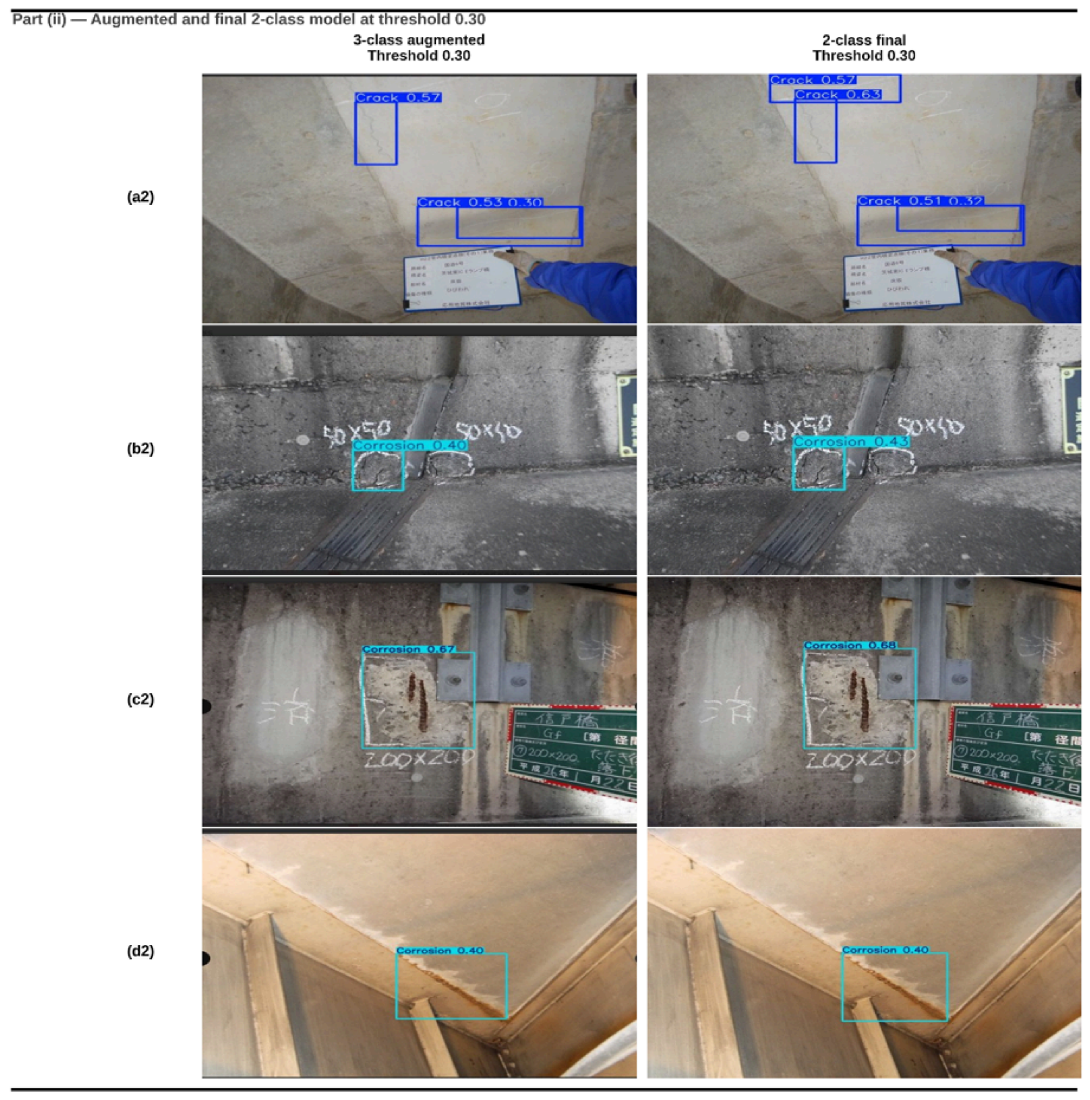
Qualitative comparison of damage detection outputs across model configurations and confidence thresholds. Part (i) shows the three-class baseline model evaluated at confidence thresholds of 0.15, 0.30, and 0.50 (Columns 1–3). Part (ii) shows the three-class model with augmented leakage data (Column 1) and the final two-class model (Column 2), both evaluated at a confidence threshold of 0.30. Row labels: (**a1**,**a2**) Crack detection on a concrete surface; (**b1**,**b2**) Small crack detection; (**c1**,**c2**) Corrosion detection on structural components; (**d1**,**d2**) Corrosion detection in a bearing area. Blue bounding boxes indicate crack detections, and cyan bounding boxes indicate corrosion detections. Note: Non-English markings visible in some inspection images are incidental field markings from the original Japanese bridge inspection dataset and are unrelated to the methodology, annotations, or interpretation of the results.

**Figure 4:**
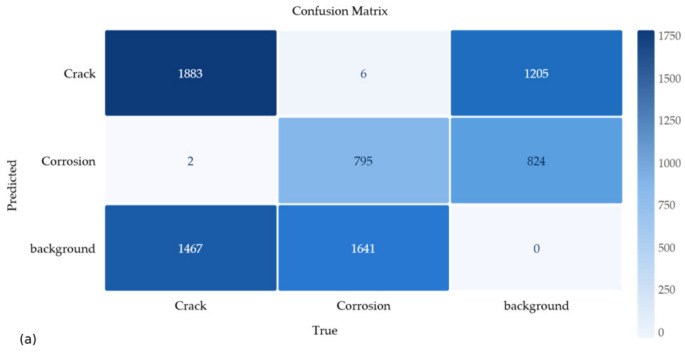

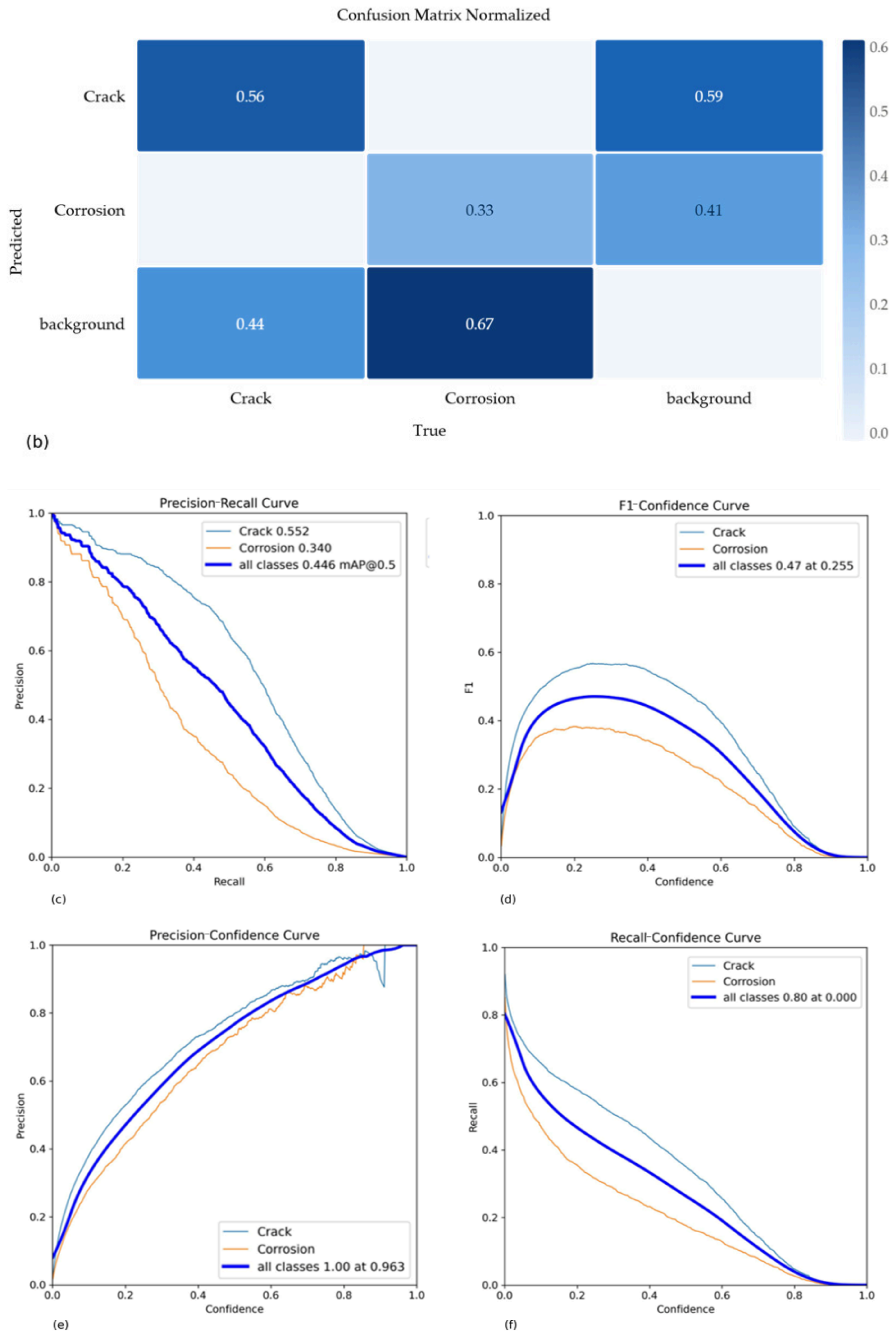
Detection performance of the final two-class YOLOv8s model. (**a**) Confusion matrix (raw counts); (**b**) normalized confusion matrix (crack missed to background: 44%; corrosion: 67%); (**c**) Precision–recall curve (Crack AP = 0.552, Corrosion AP = 0.340, mAP@0.5 = 0.446); (**d**) F1–confidence curve (peak F1 = 0.47 at confidence 0.255); (**e**) Precision–confidence curve; (**f**) Recall–confidence curve.

Based on this analysis, the leakage class was removed and a simplified two-class model was trained on crack and corrosion only. A qualitative comparison of damage detection outputs across the evaluated model configurations and confidence thresholds is presented in Figure 3. The final model achieved a Precision of 0.536, Recall of 0.427, mAP50 of 0.445, and mAP50–95 of 0.235. The corresponding F1 score was 0.474 overall (crack: 0.523; corrosion: 0.387). Class-wise mAP50 was 0.552 for crack and 0.339 for corrosion. The improvement in overall precision and mAP50 compared to the three-class baseline confirms that removing the problematic leakage class reduced class imbalance and improved detection stability. Error analysis based on the normalized confusion matrix (Figure 4b) reveals that the dominant failure mode is false negatives for low-contrast cracks and diffuse corrosion missed to background (crack: 44%; corrosion: 67%), rather than inter-class confusion between crack and corrosion (<1%). Additional failure modes include false positives from texture patterns on non-damaged surfaces misclassified as corrosion, and missed small-area damage below the model’s effective receptive field.

The comparative damage detection performance across the three model configurations is summarized in Table 2. A qualitative evaluation of detection outputs under confidence thresholds of 0.15, 0.30, and 0.50 revealed that a threshold of 0.30 provided the best balance between detection sensitivity and false positive suppression. A threshold of 0.15 produced excessive false positives, while a threshold of 0.50 resulted in missed detections, particularly for smaller or lower-contrast damage instances. Based on this analysis, a confidence threshold of 0.30 was adopted for all subsequent inference.

Figure 3 shows representative detection outputs from the final two-class model under different confidence thresholds across multiple bridge inspection images, illustrating the effect of threshold selection on detection behavior.

Figure 4 presents the detection performance evaluation plots for the final two-class YOLOv8s model, including the confusion matrix, normalized confusion matrix, precision–recall curve, F1–confidence curve, precision–confidence curve, and recall–confidence curve.

### 4.3. Structure-Aware Damage Mapping Results

The final stage of the proposed framework evaluated the ability of the integrated pipeline to associate detected damage with specific bridge structural members. The spatial assignment strategy, combining region-based overlap analysis with a center-point fallback mechanism, was applied to bridge inspection images using the Phase 2 SegFormer segmentation model and the final two-class YOLOv8s detection model.

The integrated pipeline was evaluated on a manually reviewed sample of 100 bridge inspection images selected from an independent laboratory-collected bridge inspection dataset (University of Tokyo) that was not used during model development, training, validation, or testing. Prior to evaluation, images were manually selected based on image quality, lighting conditions, visibility of structural members, and the presence of at least one target damage class (crack or corrosion) to facilitate end-to-end qualitative assessment of the proposed structure-aware inspection framework. For each image, the finalized integrated pipeline was applied, and the predicted damage type and associated structural member were manually verified through visual inspection of the pipeline output. Overall, the pipeline achieved a fully correct damage detection accuracy of 70.0% and a fully correct member assignment accuracy of 62.0%. When partially correct predictions were additionally considered for qualitative analysis, the corresponding accuracies increased to 84.0% and 87.0%, respectively. Performance varied across member classes, with the main girder class achieving the highest combined accuracy for both damage detection (90.9%) and member assignment (93.9%), while the deck slab class showed the lowest performance (75.0% and 78.1%, respectively). The lower deck slab performance is attributed to the frequent presence of secondary bridge components such as drainage pipes, railings, bearing assemblies, and parapet walls in the foreground of deck slab images, which the segmentation model tends to classify as deck slab due to background dominance. To address this limitation, future work will explore multi-scale segmentation refinement and dedicated negative-sample augmentation strategies for deck slab regions to improve the model’s ability to distinguish structural slab surfaces from foreground attachments. These results demonstrate that the proposed framework provides useful structure-aware inspection outputs across all three structural member classes, with particularly strong performance for main girder damage scenarios. The results are summarized in Table 3.

For evaluation purposes, a prediction was classified as fully correct when both the detected damage type and the assigned structural member matched the manual assessment. A prediction was classified as partially correct when either the damage type or the structural member was correctly identified, but not both. Evaluation criteria: a detection is Fully Correct if the predicted damage type matches the ground-truth label and the assigned structural member matches the ground-truth member; Partially Correct if the damage type is correct but the member assignment is incorrect, or if multiple detections are returned and at least one satisfies both criteria; and Incorrect if the damage type is wrong regardless of member assignment.

The 100 images were selected from the held-out test split of the detection dataset (871 images total), stratified proportionally across all six structure-aware label categories (crack/corrosion × main girder/deck slab/abutment). No image-level overlap exists between the evaluation sample and the segmentation training, validation, or detection training datasets. The segmentation and detection training sets are also fully independent of each other: segmentation annotations were created from a separate CVAT-labeled dataset, while detection labels were derived from four Japanese national bridge inspection datasets (Shimamoto 1–4) and a laboratory-collected bridge inspection dataset (University of Tokyo). All 100 evaluation images contain both visible structural members (identifiable by the segmentation model) and at least one damage instance (detectable by the YOLOv8s model), ensuring that both components of the pipeline are exercised in every evaluated case. The images were sourced from Japanese national bridge inspection records spanning multiple bridge structures, viewpoints (close-range and wide-angle), lighting conditions (natural daylight, overcast, and shadowed), and damage severity levels (early-stage to advanced). However, the bridges represented in the test split may partially overlap with those in the training data at the bridge-structure level (i.e., different images from the same bridge); a fully bridge-level held-out evaluation across completely unseen bridge structures is acknowledged as a limitation and is planned for future work.

**Figure 5:**
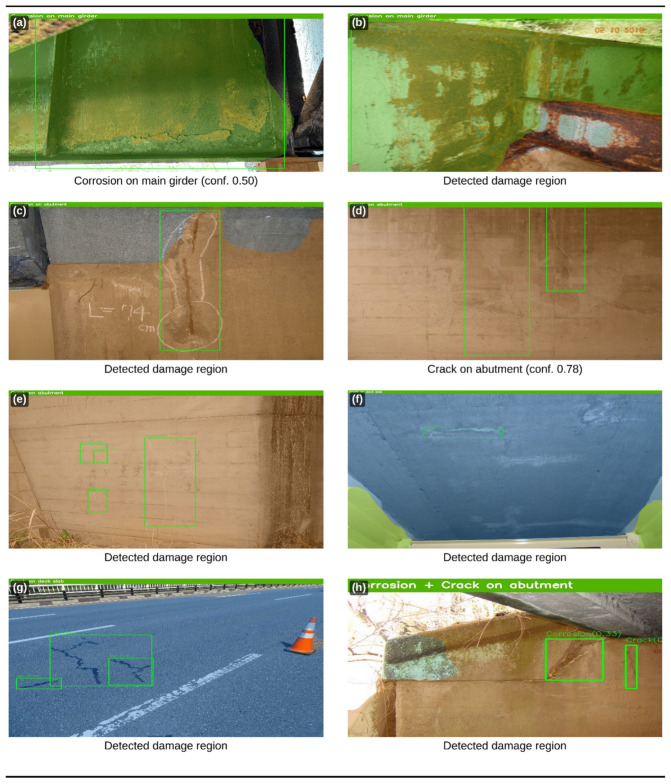
Representative structure-aware inspection outputs from the integrated pipeline across all three structural member classes and both damage types. Green bounding boxes indicate detected damage regions with predicted structural member labels and confidence scores. Panel labels: (**a**) corrosion on main girder (confidence 0.50)—single corrosion detection correctly assigned to steel main girder; (**b**) corrosion on main girder (confidence 0.73)—multiple corrosion detections on steel girder surface; (**c**) corrosion on deck slab (confidence 0.44)—corrosion correctly assigned to deck slab member; (**d**) corrosion on abutment (confidence 0.86)—high-confidence single detection correctly assigned to abutment; (**e**) crack on abutment (confidence 0.78, 0.51)—multiple crack detections correctly assigned to the same abutment member; (**f**) crack on deck slab (confidence 0.76)—multiple high-confidence crack detections on deck slab; (**g**) crack on main girder (confidence 0.62)—crack detection correctly assigned to main girder; (**h**) corrosion and crack on abutment—composite damage detection demonstrating the framework’s two-class output capability on a single structural member.

Figure 5 illustrates representative structure-aware inspection outputs from the integrated pipeline across all three structural member classes and both damage types. Figure 5a,b demonstrate corrosion detection correctly assigned to the main girder class, which achieved the highest combined accuracy in the evaluation. Figure 5c,d show correct member assignment for deck slab and abutment corrosion cases respectively, with Figure 5d achieving the highest single detection confidence of 0.86 in the illustrated examples. Figure 5e–g demonstrate crack detection across all three member classes, with Figure 5e showing multiple crack detections correctly assigned to the same abutment member. Figure 5h illustrates the framework’s composite damage labeling capability, where both corrosion and crack are simultaneously detected on a single abutment member, generating a structure-aware output of “Corrosion + Crack on abutment.” These outputs confirm that the proposed framework successfully associates detected damage with specific structural members across diverse bridge inspection imaging conditions, viewpoints, and structural configurations, providing actionable inspection information that extends beyond simple damage type classification.

## 5. Discussion

### 5.1. Segmentation Performance and the Role of SAM Refinement

The Phase 2 SegFormer model achieved a test mIoU of 0.851, representing a substantial improvement over the Phase 1 baseline of 0.611 [12]. This improvement coincided with three simultaneous methodological changes: removal of the bearing class, integration of an additional bridge member dataset to increase the training data, and the use of SAM mask-prompt refined annotations during dataset preparation. Because these changes were introduced simultaneously, the present study does not attribute the observed improvement to any single methodological component. These results are consistent with findings reported by Yu and Nishio [12], who demonstrated that accurate structural component segmentation requires sufficient class representation and precise pixel-level annotations. The substantial performance gain achieved through SAM-assisted annotation preparation highlights the value of foundation model integration in specialized dataset construction, as previously noted in studies applying SAM to domain-specific segmentation tasks [10].

The finding that SAM bounding-box prompting decreased performance (from 0.611 to 0.550) while mask-prompt refinement maintained or improved it is noteworthy. Bounding-box prompts provide only coarse spatial constraints and may produce ambiguous masks in complex structural scenes [10]. In contrast, mask-prompt mode uses the SegFormer coarse prediction as a spatial prior, guiding SAM to refine boundaries within already-identified regions. This hybrid approach leverages the complementary strengths of supervised semantic segmentation and foundation-model-based promptable segmentation [9,10], and is consistent with the broader trend of combining task-specific models with general-purpose foundation models in specialized domains. To the best of our knowledge, this is the first study to systematically compare SAM bounding-box and mask-prompt modes for structural member segmentation in bridge inspection, demonstrating that mask-prompt mode using coarse supervised predictions as spatial priors outperforms bounding-box prompting in this domain. This finding provides a practical guideline for researchers applying foundation models to specialized infrastructure inspection datasets where precise boundary delineation is critical and manual re-annotation is costly. Table 1 provides a controlled comparison to partially disentangle these effects. Phase 1 with SAM mask refinement (using the same dataset and five-class configuration, with only annotation quality modified) achieved an mIoU of 0.614 compared with the Phase 1 baseline of 0.611, indicating that SAM mask refinement alone produced only a marginal improvement. The substantially higher performance observed in Phase 2 is therefore consistent with the combined methodological changes introduced during Phase 2, including bearing-class removal, dataset expansion, and improved annotation quality through SAM mask-prompt refinement.

The deck slab class showed the largest improvement from Phase 1 to Phase 2, with IoU increasing from 0.686 to 0.802. This is likely attributable to deck slabs typically occupying large, continuous image regions that benefit most from precise boundary delineation. Main girder and abutment classes also showed consistent improvements. These findings align with observations in previous structural segmentation and inspection studies, where larger structural members with more consistent visual appearance tend to achieve higher segmentation accuracy, while small or visually ambiguous components present greater challenges for automated recognition [12,13].

### 5.2. Damage Detection Performance and Class Complexity

The damage detection results reveal an important trade-off between model completeness and detection reliability in multi-class bridge damage detection. The leakage class consistently demonstrated the weakest performance across all evaluated configurations, even after targeted augmentation. This behavior is consistent with previous studies on bridge surface damage detection, which have noted that diffuse and visually variable damage patterns—including water-related staining and leakage—are inherently more difficult to detect using bounding-box-based approaches compared to structurally compact damage types such as cracks [11].

The decision to adopt a two-class model reflects a practical engineering judgment supported by the experimental evidence: a reliable model detecting two damage types provides more consistent and actionable outputs than an unreliable three-class model. The final two-class YOLOv8s model achieved a crack mAP50 of 0.552 and an overall mAP50 of 0.445. While these values are modest compared to enhanced YOLOv8 variants specifically designed for bridge defect detection—such as BD-YOLOv8s, which reported an mAP50 of 0.862 on a dedicated bridge defect dataset [19]—direct numerical comparison is not meaningful, as BD-YOLOv8s and similar enhanced architectures were evaluated on purpose-built, curated benchmark datasets under controlled conditions. The current dataset reflects real-world inspection variability including mixed viewpoints, partial occlusion, and inconsistent lighting, which inherently reduces detection scores. The reported mAP50 of 0.445 is therefore consistent with multi-class damage detection performance on field inspection imagery [11,20]. Furthermore, the primary contribution of the present framework lies not in detection performance alone, but in the integration of detection with structural member segmentation to produce structure-aware outputs that extend beyond the capabilities of detection-only approaches. The corresponding F1 score was 0.47. A direct comparative evaluation of alternative segmentation architectures (e.g., U-Net, DeepLabv3+, Mask R-CNN) and detection models (e.g., YOLOv5s, YOLOv8n variants) on the same dataset was not conducted in the current study. Conducting full retraining experiments for multiple architectures was beyond the computational scope of this work. Such a comparative evaluation is planned as future work to strengthen architecture selection justification. The selection of SegFormer-B2 and YOLOv8s was motivated by their demonstrated state-of-the-art performance in recent bridge and infrastructure inspection literature [9,11,19].

A confidence threshold of 0.30 was selected based on the F1–confidence curve, which peaks at 0.47 near confidence 0.255. The 0.30 threshold lies just above this peak, providing near-optimal F1 while suppressing the lowest-confidence detections. The overlap threshold was refined iteratively during pipeline development. Initial thresholds of 15% and 8% were found to be overly restrictive for small localized defects, frequently resulting in unassigned detections where valid damage instances near member boundaries failed the overlap criterion. A threshold of 3% provided a more permissive assignment strategy while maintaining structural relevance and was therefore adopted for the final framework. Detections failing the overlap criterion were subsequently handled using the center-point fallback mechanism described above. However, a rigorous quantitative sensitivity analysis comparing thresholds of 1%, 3%, 5%, 8%, 10%, and 15%, together with comparisons against alternative assignment strategies (e.g., maximum-overlap, majority-label, and centroid-only assignment), was beyond the scope of this pilot-scale feasibility study. This limitation is acknowledged and will be addressed as part of the larger-scale validation study proposed for future work [21,22]. This approach is consistent with practice in object detection for structural inspection where downstream filtering provides additional quality control [18].

### 5.3. Structure-Aware Damage Mapping and Framework Integration

The integrated pipeline demonstrated the ability to generate structure-aware inspection outputs, correctly associating detected damage with structural members in 62.0% of the evaluated bridge inspection images (fully correct). When partially correct predictions were additionally considered for qualitative analysis, the corresponding value increased to 87.0%. Similarly, damage detection achieved a fully correct accuracy of 70.0%, increasing to 84.0% when partially correct predictions were included. These results demonstrate the practical feasibility of linking damage detection outputs with structural segmentation masks for automated bridge inspection, extending beyond the capabilities of existing approaches that report damage type alone without structural context [11,12,13]. The per-member-class breakdown in Table 3 reveals that the main girder class achieves the strongest performance, while deck slab presents the greatest challenge due to foreground occlusion by secondary components—a finding that motivates targeted improvements in future work.

Member assignment errors were primarily attributable to two factors: viewpoint-induced ambiguity and domain shift between training datasets. These challenges are well documented in the bridge inspection literature, where image variability across inspection conditions, viewpoints, and equipment types frequently degrades model generalization [4,5]. The use of a center-point fallback strategy partially mitigated these effects by providing a secondary assignment mechanism when region-based overlap was insufficient.

### 5.4. Practical Implications and Limitations

The proposed framework addresses a genuine gap in existing bridge inspection research by providing structure-aware outputs that support subsequent engineering assessment and maintenance planning. The ability to generate outputs such as “crack on main girder” or “corrosion on deck slab”—rather than simply “crack detected”—provides inspectors with more informative inspection outputs, consistent with the practical requirements identified in bridge maintenance guidelines [2,4]. This capability represents a meaningful step toward AI-assisted inspection systems that can support engineers in supporting subsequent maintenance assessment based on both damage type and structural location. The current results constitute a pilot-scale feasibility demonstration. Full maintenance assessment additionally requires crack length, width and orientation, corrosion extent and section loss, member criticality, structural redundancy, location along the span, and condition rating—none of which are provided by the current framework. The present work contributes the damage-to-member association layer as a necessary first step.

Several significant limitations should be explicitly noted: (1) two damage classes only (crack and corrosion); (2) three structural member classes only; (3) bearing class excluded due to class imbalance—bearings are structurally critical components (bearing class: near-zero IoU throughout training due to small spatial extent and class imbalance) and leakage exclusion reflects its high visual variability—both exclusions narrow practical deployment scope and must be understood as limitations, not optimization choices; (4) moderate detector performance (mAP50 = 0.445, F1 = 0.47); (5) pilot-scale 100-image evaluation without formal inter-rater agreement; (6) no bridge-level external validation; (7) no condition-rating or severity module; (8) SAM offline only.

## 6. Conclusions

This study demonstrated, as a pilot-scale feasibility study, that integrating transformer-based structural member segmentation, one-stage object detection, and spatial damage mapping within a unified deep learning pipeline can produce structure-aware bridge inspection outputs with 70.0% fully correct damage detection and 62.0% fully correct member assignment on 100 real bridge inspection images. When partially correct predictions were additionally considered for qualitative analysis, the corresponding accuracies increased to 84.0% and 87.0%, respectively. These results address a key limitation of existing bridge inspection approaches, which report damage type without structural context, by explicitly linking detected damage to specific structural components and thereby providing structured inspection information that can support subsequent engineering assessment and maintenance decision-making.

The following conclusions are drawn from the experimental results:

The SegFormer-based structural member segmentation model, trained using SAM mask-prompt refined annotations, achieved a test mIoU of 0.851 for three primary structural member classes—main girder, deck slab, and abutment. Compared with the Phase 1 configuration (mIoU = 0.611), the observed improvement reflects the combined effects of SAM mask-prompt refinement, bearing-class removal, and dataset refinement rather than any single modification. Because these changes were introduced simultaneously, the individual contribution of each factor cannot be isolated in the present study. Nevertheless, the results demonstrate that high-quality annotation refinement combined with appropriate dataset preparation can substantially improve structural member segmentation performance and provide practical guidance for applying foundation models to specialized bridge inspection datasets.

The YOLOv8s damage detection model trained on a simplified two-class configuration achieved a mAP50 of 0.445 for crack and corrosion detection. Although this performance was sufficient to demonstrate the feasibility of the proposed integrated framework, false-negative detections—particularly for visually subtle corrosion and fine cracks—remain the primary factor limiting downstream structure-aware damage-to-member association. The comparative evaluation across three model configurations revealed that leakage detection represents a fundamentally more challenging problem due to its intrinsic visual variability, and that reducing class complexity by removing the leakage class improved overall detection stability and precision. A confidence threshold of 0.30 was identified as optimal through qualitative threshold analysis, providing the best balance between detection sensitivity and false positive suppression.

The integrated structure-aware damage mapping pipeline successfully generated labeled outputs associating detected damage with identified structural members, achieving a fully correct member assignment accuracy of 62.0% on 100 bridge inspection images. The proposed spatial assignment strategy, combining region-based overlap analysis with a center-point fallback mechanism, proved effective in handling real inspection imagery where damage regions partially overlapped multiple structural areas. When partially correct predictions were additionally considered for qualitative analysis, the corresponding member assignment accuracy increased to 87.0%.

The proposed framework should be regarded as a first layer for AI-assisted bridge inspection that provides structure-aware damage-to-member association. Comprehensive maintenance prioritization requires additional engineering information, including damage severity, crack dimensions, corrosion extent, section loss, member criticality, and condition ratings, which are beyond the scope of the present study.

Several limitations were identified that motivate future research directions. The current framework detects only two damage types; extending coverage to spalling, delamination, and leakage remains an important next step. Although leakage detection proved challenging in the current study due to its intrinsic visual variability, future work targeting improved leakage-specific architectures and annotation strategies may improve coverage. While the evaluation on 100 images provides a meaningful initial benchmark with per-member-class breakdown, a larger-scale quantitative evaluation across diverse bridge types and inspection environments is needed to fully characterize framework performance. Furthermore, the present study did not perform bridge-level or inspection-campaign-level data separation. Consequently, the generalization capability of the proposed framework to entirely unseen bridge structures remains to be validated through future external evaluation studies.

Future work will focus on bridge-level and inspection-campaign-level external validation using entirely unseen bridge structures, together with expanding the training dataset to cover a wider range of bridge types, structural conditions, and imaging environments; incorporating additional damage categories through improved annotation strategies and class-balanced training; and developing a systematic quantitative evaluation protocol for the integrated pipeline including member assignment accuracy, damage localization precision, and framework-level performance metrics.

In particular, the detection challenges associated with visually ambiguous classes such as leakage and the structurally small bearing class motivate the application of generative data augmentation strategies. Generative adversarial network (GAN)-based image synthesis and self-training with domain adaptation, which have demonstrated effectiveness in expanding training data for rare or visually complex categories in related infrastructure inspection tasks [21,22], represent promising directions for improving detection coverage of these difficult classes without requiring extensive additional manual annotation. The integration of temporal inspection data and comparison with inspector-assigned condition ratings would further validate the practical utility of the proposed framework for real-world infrastructure monitoring applications. The framework was evaluated on steel and reinforced concrete components; applicability to timber or masonry bridges has not been validated. The framework is image-based and can potentially generalize to reinforced concrete, steel, and composite bridges provided representative training data are available. Future work will evaluate cross-material generalization through targeted dataset expansion. Structural misalignment and deformation-type damage requiring geometric or multi-frame reasoning are outside the current scope.

### Practical Implications and Limitations

The proposed framework demonstrates the feasibility of integrating structural member segmentation, automated damage detection, and spatial damage mapping to generate structure-aware bridge inspection outputs that can support maintenance planning and digital infrastructure management. As a pilot-scale feasibility study, the current framework is limited to two damage classes, three structural member classes, and evaluation using a single bridge inspection dataset. These limitations provide the basis for the future research directions discussed above.

Overall, this pilot-scale feasibility study establishes an initial benchmark for structure-aware automated bridge inspection by integrating transformer-based segmentation, efficient object detection, and spatial damage mapping within a unified deep learning pipeline. The study identifies clear directions for future development, including expanded damage and structural member classes, bridge-level external validation, enhanced detector performance, and integration with condition-rating modules for comprehensive engineering decision support.

## Figures and Tables

**Table 1:** SegFormer segmentation performance across training phases.

Phase/Configuration	mIoU	Background	Main Girder	Deck Slab	Abutment
Phase 1—SegFormer only	0.611	0.869	0.757	0.686	0.741
Phase 1 + SAM bbox refinement	0.550	0.789	0.698	0.644	0.617
Phase 1 + SAM mask refinement	0.614	0.869	0.762	0.694	0.743
Phase 2—SAM mask (bearing removed)	0.851	0.917	0.843	0.802	0.843

**Table 2:** Comparison of damage detection model configurations.

Model	Classes	Precision	Recall	mAP50	mAP50-95
3-class baseline	Crack, Corrosion, Leakage	0.482	0.423	0.409	0.212
3-class + augmented leakage	Crack, Corrosion, Leakage	0.503	0.400	0.397	0.204
2-class (final)	Crack, Corrosion	0.536	0.427	0.445	0.235

**Table 3:** Manual evaluation of the structure-aware integrated pipeline on 100 bridge inspection images. Damage Correct and Member Correct refer to the proportion of images in which the predicted damage type and structural member assignment were judged fully correct, partially correct, or incorrect upon visual inspection.

Member Class	*n*	Damage Correct (%)	Damage Partial (%)	Damage Incorrect (%)	Member Correct (%)	Member Partial (%)	Member Incorrect (%)
Main girder	33	75.8	15.2	9.1	63.6	30.3	6.1
Deck slab	32	62.5	12.5	25.0	53.1	25.0	21.9
Abutment	35	71.4	14.3	14.3	68.6	20.0	11.4
Overall	100	70.0	14.0	16.0	62.0	25.0	13.0

Yes + Partial combined accuracy: Damage = 84%; Member = 87%.

## Data Availability

The data presented in this study are available on request from the corresponding author.
